# Calcifying pseudoneoplasm of the neuraxis (CaPNoN): an unusual cause of third nerve palsy in a teenager

**DOI:** 10.1259/bjrcr.20150494

**Published:** 2016-07-28

**Authors:** Joseph Ghaemi, Mohammed Wasimi, Rekha Siripurapu, David McKee, Piyali Pal, Daniel du Plessis, Scott Rutherford, Roger Laitt, Gillian Potter

**Affiliations:** ^1^Department of Neuroradiology, Greater Manchester Neurosciences Centre, Salford Royal NHS Foundation Trust, UK; ^2^Department of Neurology, Greater Manchester Neurosciences Centre, Salford Royal NHS Foundation Trust, UK; ^3^Department of Pathology, Greater Manchester Neurosciences Centre, Salford Royal NHS Foundation Trust, UK; ^4^Department of Neurosurgery, Greater Manchester Neurosciences Centre, Salford Royal NHS Foundation Trust, UK

## Abstract

An 18-year-old part-time teacher presented with headache and diplopia. Physical examination showed partial left oculomotor palsy. Neurology examination was otherwise unremarkable. Cross-sectional imaging was arranged for investigation of third nerve palsy. On CT scan, the lesion was calcified, and on MRI, hypointense on *T*_1_ and *T*_2_ weightedimages with thin rim enhancement, resembling an atypical meningioma. CT angiogram showed no vascular connection. Following worsening diplopia and a slight increase in lesion size on follow-up MRI, the patient was re-reviewed in our regional skull base multidisciplinary team meeting, where a decision for excision was made. Pre-operatively, the absence of a vascular connection was confirmed on catheter angiogram. Histopathological examination demonstrated features typical of calcified pseudoneoplasm of the neuraxis, with extensive metaplastic calcification with stroma containing variable fibrovascular tissue and focal inflammatory cell infiltrates, spindle and epithelioid cells, and psammoma bodies at the rim of the lesion. Following surgery, the patient had persisting diplopia. He remains under clinical review. As surgical resection is considered curative, no further imaging follow-up is planned.

## Clinical presentation

An 18-year-old part-time teacher presented with headache and diplopia. Headache was intermittent and side-locked, affecting the left temporal region, lasting up to 4 h at a time over a 2-year period, with a gradual worsening in frequency and severity and accompanied by new intermittent diplopia. The headache was refractory to analgesics. There were no other cranial nerve symptoms. Physical examination showed a partial left oculomotor palsy with partial limitation of downgaze, adduction of the eye and sluggish left pupillary response. There was no ptosis. Neurological examination was otherwise unremarkable.

## Imaging findings

**Figure 1.**
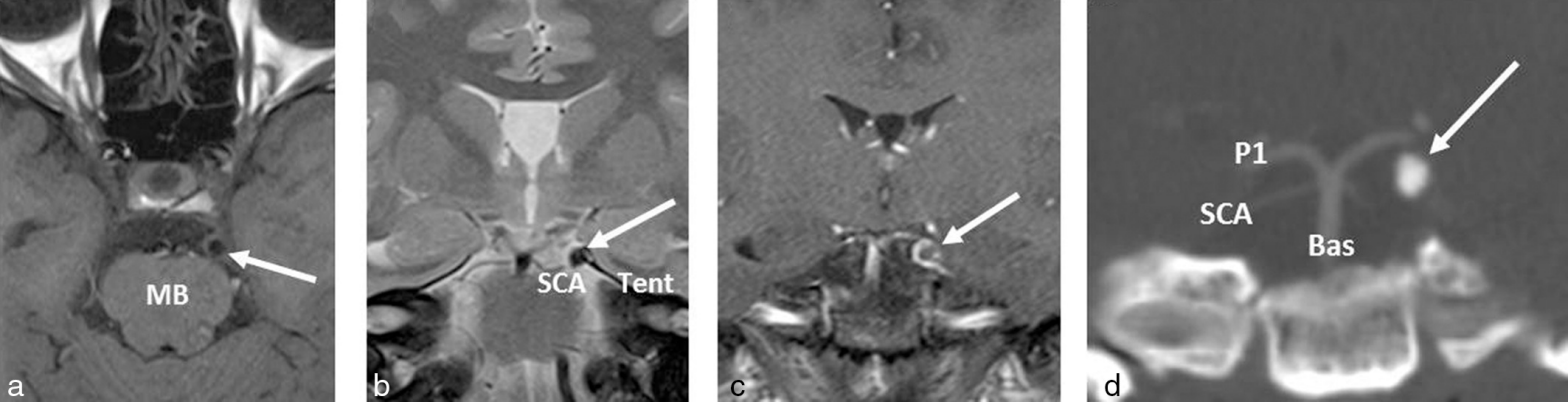
Axial *T*_1_ (a) and coronal *T*_2_ weighted fat-saturated (b) MRI showing a well-defined, small, rounded, hypointense lesion (arrows) in close relationship to the left tentorium (Tent) and left SCA at the level of the MB. The lesion (arrow) demonstrates thin peripheral enhancement on coronal *T*_1_ weighted fat-saturated post-contrast imaging (c). On coronal maximum intensity projection reconstructions of CT angiogram of circle of Willis (d), the lesion (arrow) demonstrates density consistent with calcification and lies in the expected location of the proximal left cisternal third nerve, between the SCA and P1 segment of the left posterior cerebral artery, with no vascular connection, which was also confirmed on catheter angiogram (images not shown). Bas, basilar artery; MB, midbrain; SCA, superior cerebellar artery.

MRI of the skull base was performed to investigate the left third nerve symptoms and signs and demonstrated a *T*_1_ and *T*_2_ hypointense mass in the expected location of the proximal left third nerve, between the P1 segment of the left posterior cerebral and the left superior cerebellar arteries (Figure 1a,b). There was minor peripheral enhancement (Figure 1c). CT angiogram of the circle of Willis demonstrated a calcified lesion with no vascular connection (Figure 1d), which was also confirmed on catheter angiogram. Differential diagnosis included heavily calcified meningioma and calcifying pseudoneoplasm of the neuraxis (CaPNoN).

## Outcome, follow-up and discussion

**Figure 2.**
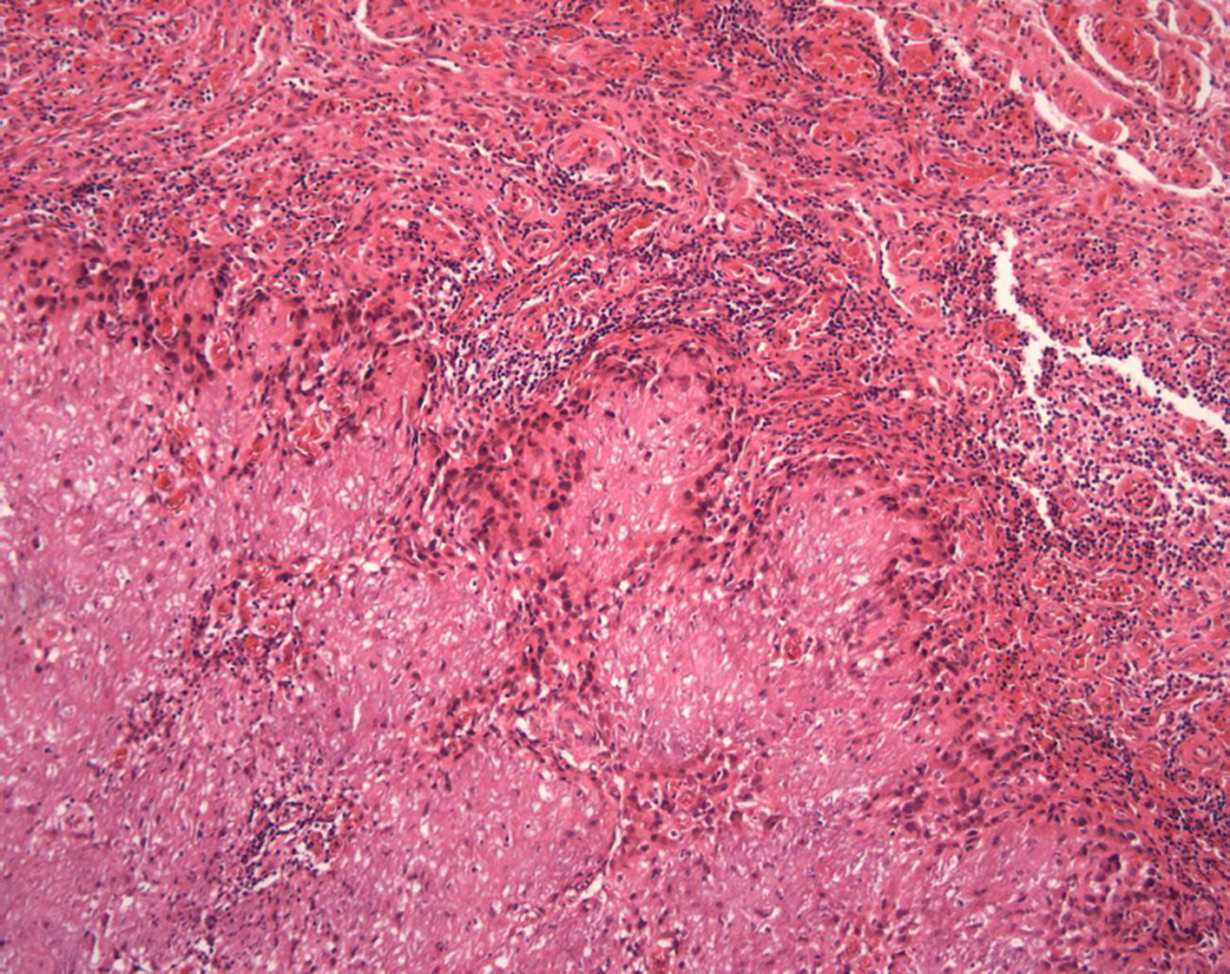
Focally calcified amorphous chondromyxoid matrix (bottom and left) surrounded by a rim of epithelioid to spindle cells (epithelial membrane antigen immunopositivity not shown) and separated from the brain by a band of granulation type tissue (top and right) (haematoxylin and eosin stain, original magnification ×100).

Diplopia was progressive and follow-up MRI demonstrated a slight but definite growth of the lesion, consistent with progressive diplopia. Following a multidisciplinary team discussion, the lesion was resected using a subtemporal approach. It had arisen from the free edge of the tentorium and was not adherent to the brainstem or blood vessels. The tentorium was divided around the lesion to allow en bloc removal. Histopathological examination demonstrated extensive metaplastic calcification with stroma containing variable fibrovascular tissue, with focal inflammatory cell infiltrates, spindle and epithelioid cells, and psammoma bodies at the rim of the lesion showed positive staining for epithelial membrane antigen (Figure 2). There were no meningothelial cells. Staining for β-amyloid was negative.

CaPNoNs, first described in 1978,^1^ are rare, benign lesions, possibly reactive, which may occur anywhere in the central nervous system. They may be intra- or extra-axial in location. Previous case reports of intracranial CaPNoN, the majority of which have been extra-axial,^2^ have been associated with various symptoms depending on the site, including headache, facial pain, cranial nerve paralysis, developmental delay and seizures, related to local irritation or compression of adjacent tissues. To our knowledge, this is the first case of CaPNoN causing third nerve palsy. Aiken et al^2^ describe in detail the imaging features of CaPNoNs; however, there are relatively few reports of this entity in the imaging literature, with most reports occurring in the neurosurgery and pathology literature.^3,4^ Histopathology typically demonstrates variable amounts of fibrous stroma, spindle to epithelioid cells, ossifications, foreign body giant cells and occasional psammoma bodies. On imaging, and depending on the location, these lesions may be misdiagnosed as calcified neoplasms such as ganglioglioma and oligodendroglioma, vascular lesions such as cavernous haemangiomas, and dural lesions such as heavily calcified meningioma. CT scan typically demonstrates calcification, and on MRI, these lesions are typically hypointense on both *T*_1_ and *T*_2_weighted imaging with minimal linear internal or rim enhancement.^2^ Larger lesions may show serpiginous internal enhancement, hypothesized to represent the vascular or stromal characteristics.^2^ Complete surgical resection is considered curative.^3^

## Learning points

1. CaPNoN is a rare, benign lesion, possibly reactive, which may occur anywhere in the central nervous system.
2. On MRI, a calcifying pseudoneoplasm appears as a *T*_1_ and *T*_2_ weighted hypointense lesion with minimal linear internal or rim enhancement and demonstrates calcification on CT scan.
3. In the presence of a calcified intracranial lesion, the diagnosis of CaPNoN should be considered in addition to the more commonly seen calcified lesions such as calcified meningioma, cavernous malformation and calcified neoplasm.

## Consent

Written informed consent for the case to be published (including images, case history and data) was obtained from the patient for publication of this case report.
